# Explosive Quadriceps Strength and Landing Mechanics in Females with and without Anterior Cruciate Ligament Reconstruction

**DOI:** 10.3390/ijerph17207431

**Published:** 2020-10-13

**Authors:** Yu-Lun Huang, Eunwook Chang, Samuel T. Johnson, Christine D. Pollard, Mark A. Hoffman, Marc F. Norcross

**Affiliations:** 1 Department of Kinesiology, College of Education and Human Sciences, University of Wisconsin–Eau Claire, Eau Claire, WI 54702, USA; 2 Department of Kinesiology, Inha University, Incheon 22212, Korea; 3 School of Biological and Population Health Sciences, College of Public Health and Human Sciences, Oregon State University, Corvallis, OR 97331, USA; Sam.Johnson@oregonstate.edu (S.T.J.); mark.hoffman@oregonstate.edu (M.A.H.); marc.norcross@oregonstate.edu (M.F.N.); 4 School of Biological and Population Health Sciences, College of Public Health and Human Sciences, Oregon State University-Cascades, Bend, OR 97701, USA; christine.pollard@osucascades.edu

**Keywords:** loading rate, osteoarthritis, quadriceps function

## Abstract

Lower explosive quadriceps strength, quantified as rate of torque development (RTD), may contribute to landing mechanics associated with anterior cruciate ligament (ACL) injury risk. However, the association between quadriceps RTD and landing mechanics during high demand tasks remains unclear. Therefore, this study investigated the influence of quadriceps RTD on sagittal plane landing mechanics during double-leg jump landings (DLJL) and single-leg jump cuts (SLJC) in females with and without ACL reconstruction (ACLR). Quadriceps RTD was measured during isometric muscle contractions. Landing mechanics were collected during DLJL and SLJC tasks. Separate stepwise multiple linear regression models determined the amount of variance in sagittal plane landing mechanics that could be explained by quadriceps RTD, group (ACLR or Control), and their interaction. The results indicate that greater quadriceps RTD is associated with lower loading rate (*p* = 0.02) and longer time to peak vertical ground reaction force (*p* = 0.001) during SLJC, regardless of ACLR status. As greater loading rate may lead to higher risk of ACL injuries and post-traumatic knee osteoarthritis post-ACLR, explosive muscle strength interventions might be useful for individuals with and without ACLR to facilitate the use of safer landing mechanics.

## 1. Introduction

Anterior cruciate ligament (ACL) injury, a devastating injury in sport, frequently occurs among individuals who participate in cutting and landing activities [1,2]. Approximately 250,000 ACL injuries have been reported annually in the United States with an estimated 50% of these occurring in the young athletic population [3,4,5]. Even though ACL reconstruction (ACLR) is considered the gold standard treatment for athletes who wish to return to high-level activities after ACL injury, ACLR alone, unfortunately, does not guarantee a reduced risk of subsequent injuries after return to play. In fact, a high second ACL injury rate has been reported following ACLR [6,7]. Compared with individuals without a history of ACL injury, the risk of a second ACL injury on either the involved or uninvolved side is higher in athletes with ACLR within two years following return to sport [6,7]. Moreover, the risk of future osteoarthritis development [8] that is associated with a higher impact loading rate [9] has not been shown to be reduced by ACLR surgery. A meta-analysis reported that the prevalence of knee osteoarthritis at 5, 10 and 20 years post-ACLR was 11.3% (6.4–19.1%), 20.6% (14.9–27.7%), and 51.6% (29.1–73.5%), respectively [10]. Therefore, it is important to identify modifiable risk factors to minimize the risk of primary and second ACL injury, as well as other long-term health complications post-ACLR.

Altered sagittal plane landing mechanics in the involved limb following ACLR including landing with less peak knee flexion and internal knee extension moment during a single-leg hop for distance has been observed, compared with individuals without ACLR [11]. Such lower extremity biomechanics alterations have been linked to quadriceps function deficits that commonly occur post-ACLR [12,13]. Lower quadriceps muscle strength is associated with lower knee flexion at initial contact (IC) during single-legged stop-jump task [14]. It is likely that this reduction in knee extension moment on the ACLR limb is driven by the use of a more extended knee position during landing to compensate for the weak quadriceps muscle [15]. However, the use of a more extended knee position under a given quadriceps muscle contraction elevates ACL injury risk [16]. Therefore, developing sufficient quadriceps muscle strength may be essential for safer landing mechanics.

Since quadriceps muscles are the primary active stabilizers of the knee joint during dynamic tasks, evaluating and developing greater quadriceps peak strength has been emphasized in current post-ACLR rehabilitation. However, the peak torque generated by the quadriceps muscles during isometric contraction does not occur until 250–300 ms after contraction onset [17]. Therefore, being able to generate sufficient forces within the critical timeframe (100 ms after IC) of the occurrence of ACL injury and the greatest ACL strain that corresponds to the peak GRF [18,19] may lend important insights into correcting the altered landing mechanics following ACLR.

Deficits in quadriceps muscle function have been identified in individuals post-ACLR including reduction in peak muscle strength and explosive strength, quantified as rate of torque development (RTD), particularly in the early phase of the contraction (<100 ms) [20]. Moreover, the recovery rate of explosive quadriceps muscle function is slower than that of peak muscle strength [21]. This timeframe coincides with the critical timeframe of the occurrence of ACL injury and the greatest ACL strain that are proposed to occur within 50 ms after IC, which corresponds to the peak GRF (0–88 ms) [18,19]. During this critical injury timeframe, lacking sufficient explosive quadriceps strength following ACLR may compromise an individual’s capacity to generate a sufficient knee extension moment. Consequently, it may further impact the use of safer landing mechanics consisting of a greater knee flexed position utilized to control for greater center of mass deceleration [15].

The notion of the potential relationship between quadriceps RTD and landing mechanics has been explored in previous studies examining walking and running mechanics in individuals post-ACLR. Greater quadriceps RTD was found to be associated with less peak vertical ground reaction force (vGRF) and loading rate during walking [22]. Additionally, Kristin et al. [12] found that individuals six months post-ACLR with lower quadriceps RTD demonstrated smaller knee flexion excursion and lower rate of knee extension moment in the injured limb when compared with the uninvolved limb during running. The findings of these studies [12,22] indicate that individuals post-ACLR with lower quadriceps RTD are unable to produce a knee extension moment quickly enough to respond to the impact force, and may result in the alterations of sagittal plane landing mechanics during walking and running. However, few studies have investigated the influence of quadriceps RTD on landing mechanics during high demand activities involving jump-landing and cutting maneuvers that better reflect the demand incurred during sports. Therefore, the purpose of this study was to investigate the influence of quadriceps RTD on sagittal plane landing mechanics in females with and without ACLR during high demand landing and cutting tasks. We hypothesized that greater quadriceps RTD would be associated with landing mechanics suggestive of a reduced risk of ACL injury.

## 2. Experimental Section

### 2.1. Patients

This study was a cross-sectional laboratory study. The study protocol was approved by the University’s Institutional Review Board (Study ID: 7000). All participants completed a written informed consent before the beginning of data collection.

Thirty-five recreationally active females (19 females post-ACLR and 16 females without ACL injury and ACLR) defined as participating in moderate to vigorous physical activity at least 150 min weekly [23] and participating in cutting or jumping sports for ≥2 sport seasons volunteered to participate in this study. The participant eligibility criteria and study participants were the same as our previous work [24,25]. Females post-ACLR were eligible if they had a unilateral ACLR within the past 1 to 5 years and had received medical clearance for unrestricted activity by their orthopedic surgeon. Participants were excluded if they reported a history of back and lower extremity surgery and injury within the 6 months from the study participation, a neurological or cardiopulmonary disorder that was diagnosed by a physician, or a history of graft failure after ACLR or multiple ACLRs. The International Knee Documentation Committee 2000 (IKDC 2000) subjective knee evaluation forms and the Knee Outcome Survey Activities of Daily Living Scales (KOS-ADLS) were utilized to evaluate the subjective knee function. Participants were excluded if their scores for question number 7 was less than 3, any item in question number 9 was less than 2 on the IKDC 2000, or any score was less than 3 on any questions on the KOS-ADLS. According to the participant eligibility criteria, five participants were excluded. Tegner Activity Scale was used to assessing activity level. A total of thirty females, 18 females post-ACLR and 12 females without ACLR, completed this study (Table 1). Our ACLR and control participants reported on average 6.4 and 6.6 on the Tegner Activity Scale, respectively. Such findings indicate that our participants were recreationally physically active and involved in medium to high demand sports such as soccer, basketball, and handball.

### 2.2. Landing Biomechanics Analysis

For landing biomechanics testing, participants wore their own athletic shoes, spandex shorts and shirt. Standardized footwear was not used to maximize the study’s generalizability and minimize potential errors in that the use of an unfamiliar shoe could affect the nature landing mechanics of the participant [26]. This approach of not controlling for footwear has also been used in multiple previous studies [27,28,29]. Each participant was asked to perform 5 min warm-up protocol on a stationary bicycle at a submaximal intensity. A standard retroreflective marker set was attached bilaterally by the same researcher over anatomical landmarks, consisting of the 1st and 5th metatarsal heads, heel counter of the shoe, medial and lateral malleoli, anteromedial tibia, medial and lateral femoral epicondyles, anterior thigh, greater trochanter, anterior superior iliac spine, posterior superior iliac spine and the acromion process. In addition, a marker was placed on the space between the 5th lumbar and 1st sacral spinous processes and over the jugular notch where the clavicles meet the sternum. After a static calibration trial, the markers on medial knee and ankle were removed. Only the surgical limb of the ACLR group and non-dominant limb of healthy group were analyzed. Leg dominance was determined by the following 3 tasks: stepping up onto a small step, kicking a ball for distance, and recovering from a small perturbation from behind [30]. The leg used to complete 2 out of 3 tasks was identified as the dominant lower limb.

A 9-camera motion capture system (Vicon, Inc., Lake Forest, CA, USA) interfaced with two force plates (Bertec Corp., Columbus, OH, USA) were used to collect landing biomechanics data. Participants required to perform three successful trials of both a double-leg jump landing (DLJL) and single-leg jump cut (SLJC). At least 3 practice trials were performed by each participant for familiarization with the tasks and set-up. For DLJL, participants were instructed to jump forward off a 30-cm box placed with the distance of 50% of the participant’s height away from the force plates [31], and perform a double-leg landing, with each foot contacting one of the force plates, and immediately jump vertically for greatest height. Successful trials were defined as participants jumped from the 30-cm box and landed on the force plates with two feet simultaneously and completed a vertical jump without any foot slipping during the landing.

For the SLJC, participants stood at a distance equivalent to 50% of their height away from the force plates. A 17 cm hurdle was placed between their standing position and the force plates. They were asked to jump over the hurdle with two legs and then land on the force plate with one foot and immediately cut at a 60 degree angle to the opposite direction from the testing limb. For example, when the testing limb was their left leg, the participant would land on the left leg, pushing off and immediately cutting to the right side [32]. Trials were considered successful if the participant jumped over the hurdle with two legs, landed with the testing leg on the force plate entirely, and cut immediately in the opposite direction from the testing leg.

### 2.3. Rate of Torque Development Measurement

Quadriceps RTD was collected on a Biodex System 3 dynamometer (Biodex Medical Systems, Inc., Shirley, NY, USA) in a sitting position with the trunk inclined 70° from the horizontal and the knee flexed to 70°. The lateral femoral condyle of the testing thigh was aligned to the axis of rotation of the dynamometer. Straps were used to minimize any unwanted movement and compensation. Participants were instructed to extend their knee against the dynamometer by isometrically contracting the quadriceps muscle as hard and fast as possible with arms across the chest. At least 2 practice trials were provided to each participant for familiarization. Verbal encouragement during the testing was provided. Three successful trials were collected; defined as when there was no initial countermovement and at least 2–3 s of a maximal plateau on the torque-time curve was observed. To minimize any effect of fatigue, a one-minute break between trials was provided.

### 2.4. Data Reduction and Analysis

Kinematic and kinetic data were sampled at 120 Hz and 1560 Hz, respectively. The raw three-dimensional (3-D) coordinates of the reflective markers during each landing task were labeled. A macro was utilized to predict missing reflective markers’ location for segments with ≥4 markers (BodyBuilder, Vicon, Lake Forest, CA, USA). The MotionMonitor software (Innovative Sports Training, Inc., Chicago, IL, USA) was used to analyze the 3-D coordinates of the reflective markers and force plate data. The hip joint center was estimated by identifying the anterior superior iliac supine marker location according to the Bell method [33]. The ankle joint centers were determined as the middle points between medial and lateral malleoli. The knee joint centers were determined as the middle points between medial and lateral epicondyles. The shank, thigh, and pelvis local coordinate systems were defined with the positive x, y, and z axes directed anteriorly, to the left and superiorly, respectively. The kinematics data were flittered using a 4th order low-pass Butterworth filter at 12 Hz, and then re-sampled at 1560 Hz and time-synchronized to the kinetic data using cubic spline interpolation method. The knee joint positions were calculated based on a right-hand conventions using Euler angles in a y-x-z sequence. The force plate data were also filtered with a 4th order low-pass Butterworth filter at 12 Hz [34]. Internal joint moments, anterior tibial shear force (ATSF), and other kinetic data were calculated by using force place data, kinematics, and anthropometric data with an inverse dynamics approach in The MotionMonitor software. The time when the vGRF >10 Newton was identified as initial contact by using custom computer software (LabVIEW, National Instruments, Austin, TX, USA). LabVIEW was also utilized to identify the kinematic variables of interest: knee flexion at IC, and knee flexion excursion and peak knee flexion during the landing phase (IC to peak knee flexion), and the kinetic variables of interest: peak knee extension moment, peak vGRF, peak posterior ground reaction force (pGRF) and peak ATSF during the initial 100 ms after IC, and time to these peak kinetics. Knee flexion excursion was calculated by subtracting peak knee flexion from knee flexion at IC. The peak knee extension moment, peak vGRF, peak pGRF, and peak ATSF were normalized to body weight (× N^−1^). The rate of knee extension moment and loading rate of vGRF, pGRF and ATSF were calculated as the peak kinetics divided by the time to reach the corresponding peak kinetics. An average of the three trials for all the kinematic and kinetic variables for each landing task were used for further statistical analysis

The raw voltage signal from the Biodex System 3 dynamometer was collected with a Biopac MP100 data collection system sampled at 1000 Hz (Biopac Systems Inc., Goleta, CA, USA). LabVIEW was utilized to analyze data. A 4th order low-pass Butterworth filter was used to filter the torque signals with a cutoff frequency of 10 Hz. Quadriceps RTD was calculated by fitting a line of best fit to the recorded torque-time curve between torque onset (i.e., defined as the point when torque exceeded 2.5% of the peak torque of that trial) [17] and 100 ms after onset and normalized by body mass (× kg^−1^) [35]. The trial with the maximum quadriceps RTD was identified for statistical analysis.

### 2.5. Statistical Analysis

Participant demographic information and quadriceps RTD were compared between ACLR and control participants using independent-sample *t*-tests. We fitted separate stepwise multiple linear regression models to determine the amount of variance in each of the dependent variables for DLJL and SLJC (DVs: knee flexion at IC, knee flexion excursion, peak knee flexion, peak knee extension moment, peak vGRF, peak pGRF, peak ATSF, time to peak knee extension moment, time to peak vGRF, time to peak pGRF, time to peak ATSF, the rate of knee extension moment, and loading rate) that could be explained by Group, RTD, and their interaction using the following equation:

DV = β0 + β1 (RTD) + β2 (Group) + β3 (RTD × Group)(1)

All statistical analyses were performed using commercially available statistical software (SPSS 24.0, IBM Corp. Armonk, NY, USA) with a priori statistical significance set at α ≤ 0.05.

## 3. Results

**Figure 1:**
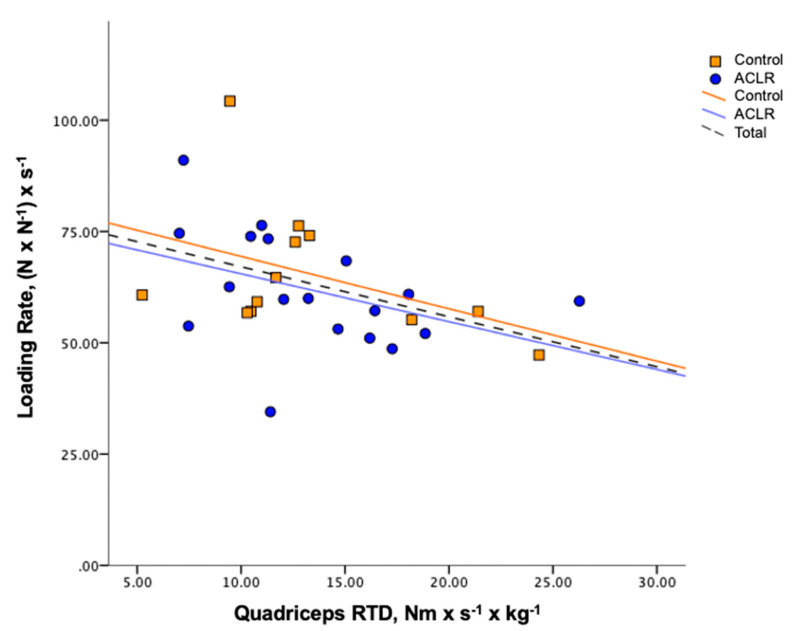
Group scatter and regression lines of loading rate by quadriceps rate of torque development (RTD) during single-leg jump cut.

**Figure 2:**
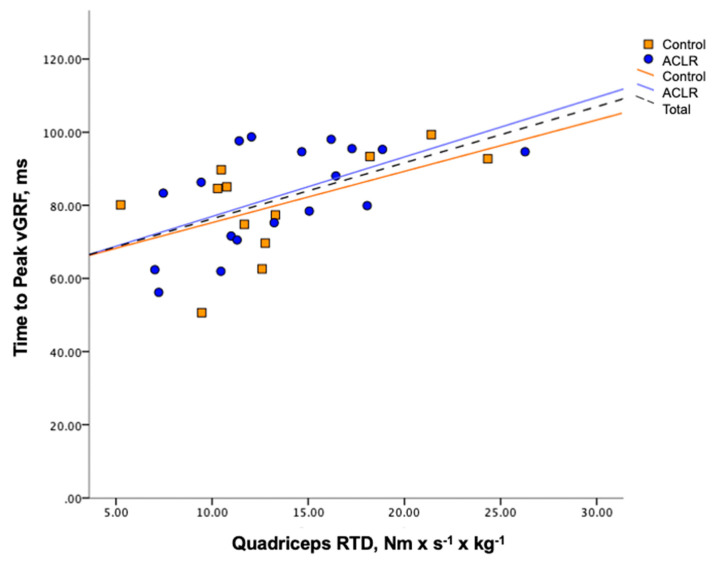
Group scatter and regression lines of time to peak vertical ground reaction force (vGRF) by quadriceps rate of torque development (RTD) during single-leg jump cut.

The variance in quadriceps RTD significantly predicted 17% of the variance in the loading rate (Model: loading rate = 78.31 − 1.12 (RTD), *p* = 0.02, Figure 1) and 31% of the variance in the time to peak vGRF (Model: time to peak vGRF = 60.95 + 1.54 (RTD), *p* = 0.001, Figure 2) during SLJC. These results indicate that greater quadriceps RTD was predictive of a lower loading rate and longer time to peak vGRF, regardless of whether or not participants had a previous ACLR. No biomechanical variables of interest during DLJL were significantly predicted by Group, RTD, or their interaction. No group differences in any landing mechanics variables was found according to the result of the regression analyses. Table 2 and Table 3 summarize the landing biomechanics during DLJL and SLJC, respectively.

## 4. Discussion

The purpose of this study was to investigate the influence of quadriceps RTD on sagittal plane landing mechanics during high demand landing and cutting tasks. The primary findings of this study indicate that greater quadriceps RTD is associated with a lower loading rate and longer time to peak vGRF during SLJC regardless of ACLR status. Contrary to our hypotheses, during DLJL, none of the biomechanics of interest were significantly predicted by ACLR status, quadriceps RTD, or their interaction.

Generating enough knee extension moment via quadriceps contraction during a high demand task is essential to allow for safer landing mechanics consisting of a greater knee flexed position to control for greater center of mass deceleration [15]. However, quadriceps muscle function deficiency is commonly observed post-ACLR [20]. This prolonged quadriceps dysfunction may lead to a higher risk of second ACL injury [6,7], and long-term joint complications [36]. Furthermore, quadriceps muscle weakness and reduction in internal knee extension moments after ACL injury may negatively affect the capacity of the quadriceps for energy attenuation, which has been suggested as a possible contributor to accelerated development of post-traumatic osteoarthritis [37,38,39].

Altered lower extremity biomechanics have been associated with an increased risk of post-ACLR knee osteoarthritis [40,41]. It has been shown that insufficient quadriceps contraction at heel strike is associated with higher impact forces [36]. Articular cartilage is susceptible to a higher rate of loading due to its viscoelastic property that leads to an elevated risk for tissue breakdown [9]. Additionally, the results from an animal study shows that, regardless of the magnitude of the load, repetitive high rate loading accelerates degeneration of the articular cartilage in rabbit knees [42]. Thus, a higher loading rate during landings may place one at greater risk for the development of knee osteoarthritis. Our findings provide evidence to support that females who had greater quadriceps RTD also demonstrated lower loading rate and longer time to peak vGRF during SLJC regardless of ACLR status. Such results indicate that improving explosive quadriceps strength may reduce the risk of knee osteoarthritis development in females with and without ACLR. Our findings are of particular importance because we identified the protective effect of greater quadriceps RTD exists not only in females post-ACLR, but also in females without a history of ACLR. These data highlight the potential importance of incorporating quadriceps explosive strengthening interventions to enable more favorable landing mechanics with respect to lower extremity injury risk regardless the ACLR status.

In our study, the loading rate during the first 100 ms after IC is calculated as the normalized peak vGRF divided by the time to reach peak vGRF within this time interval. Interestingly, we identified that the lower loading rate associated with greater quadriceps RTD is primarily driven by the longer time to peak vGRF. Moreover, the lower loading rate, in fact, had little to do with the magnitude of peak vGRF since peak vGRF was not predicted by quadriceps RTD. Landing with higher loading rates that are the result of shortened time to peak vGRF makes it more challenging to dissipate the GRF compared to the same magnitude of peak vGRF reached at a later time. Thus, greater quadriceps RTD may enable individuals to minimize the negative impact of loading rate during landing achieved through a longer time to peak vGRF, which potentially may prevent knee osteoarthritis for females with and without ACLR.

In addition to the potential protective effect of greater quadriceps RTD on chronic lower extremity injuries, having greater quadriceps RTD may also be protective against a second ACL injury. Hewett et al. [43] found that uninjured female athletes who went on to suffer a non-contact ACL injury demonstrated a 16% shorter stance time during landing than female athletes who did not suffer an ACL injury. Given that absorbing force over shorter time duration is a factor known to be associated with the risk of ACL injury [43], developing greater explosive quadriceps strength may also prevent primary and second ACL injuries by lengthening the time to peak vGRF, which may allow for the use of a less stiff landing strategy. As such, future studies identifying interventions targeting explosive quadriceps strength applicable for ACL injury prevention programs and post-ACLR rehabilitations are needed.

According to our regression analysis, none of the landing biomechanics variables of interest during both tasks were predicted by group. This finding indicates that no group difference in any landing mechanics variables was found. These findings were unexpected because alterations in landing mechanics [12,13,22,44] compared with the uninvolved limb or heathy controls have been reported in previous studies. In addition, the deficits in the capacity to rapidly generate quadriceps muscle forces exist both isometrically and dynamically, quantified as rate of knee extension moment during running, 6 months after ACLR [12]. A potential explanation of these unexpected finding is that our ACLR participants were on average 35.1 ± 13.7 months after ACLR with no difference in quadriceps RTD compared with our control group (Table 1). Our findings may indicate that, as quadriceps RTD in females post-ACLR is similar to their healthy counterparts, their landing mechanics would be also similar to their healthy counterpart.

Relevant studies have commonly used individuals without a history of ACLR as a reference of sufficient quadriceps function and safer landing mechanics since the ACL injury risk increases significantly in individuals post-ACLR compared with individuals who have never injured their ACLs [6,45]. Even though there is general agreement that individuals who have never injured their ACL have normal and safer landing mechanics, this assumption needs to be used carefully due to the existing variability in quadriceps function in individuals without ACLR. Overall, both ACLR and control groups in our study had quadriceps RTD on average higher than 13 Nm × s^−1^ × kg^−1^ (Table 1), which, as a group, had high quadriceps RTD performance. However, the variability of quadriceps RTD in individuals with and without ACLR—with a spread between low and high quadriceps RTD—may result in great variability in their landing mechanics profiles. This notion is, in fact, evidenced by the results of our study that indicates that greater RTD is associated with lower loading rate and time to peak vGRF regardless the ACLR status. It may suggest that, regardless the ACLR status, improving quadriceps RTD, even in individuals with a relatively high explosive quadriceps strength performance, may continuously improve the use of a safer landing mechanics with respect to ACL injury risk and long-term joint health.

In a previous study, individuals post-ACLR, with an average four years following ACLR, with quadriceps RTD deficient demonstrated lower knee extension moment and rate of knee extension moment, and higher loading rate during running on their ACLR limbs, compared with the control group [13]. Contrary to our findings, they identified a weak association between quadriceps RTD and rate of knee extension moment for the ACLR limbs, but no significant relationship between quadriceps RTD and loading rate was found [13]. The different findings in our study may be driven by the type of tasks and higher demand of the tasks. The average vGRF during SLJC in our study was approximal 3-fold of the average vGRF during running reported in Pamukoff et al. study [13]. Additionally, the average rate of knee extension moment during SLJC in the current study was approximal 2-fold of the average rate of knee extension moment during running reported in Pamukoff et al. study [13]. It has been shown that, as the demand of tasks increased while dropping from a higher height, the peak vGRF increased accompany with elevated loading rate and shorter time to peak vGRF [46]. It is possible that as the demand increased (e.g., greater vGRF and rate of knee extension moment) during SLJC performed in our study, the effect of greater quadriceps RTD on rate of knee extension moment might has been maxed up. Consequently, greater quadriceps RTD potentially could not further speed up the rate of knee extension moment anymore during high demand tasks like SLJC. Instead, the protective effect of greater quadriceps RTD during such high demand tasks was achieved through lengthening the time to peak vGRF to lower the loading rate. This may allow longer time for energy attenuation and to lower risk of lower extremity injuries. The underlying mechanics for how greater quadriceps RTD facilitates longer time to peak GRF remains unclear. Future studies are needed to investigate the underlying mechanics of the protective effect of greater explosive quadriceps strength on landing mechanics during different tasks.

Unlike SLJC, contrary to our hypotheses, no biomechanical variables of interest during DLJL were significantly predicted by quadriceps RTD. A potential explanation of this finding is that double-leg task may allow for a compensatory landing mechanism by shifting stress to the uninvolved limb or the limb with greater explosive quadriceps strength if quadriceps RTD asymmetry exist. Evaluating the quadriceps RTD magnitude of the ACLR limb or non-dominant limb in individuals without ACLR may be insufficient to capture compensatory landing mechanism during double-leg task. This notion was supported by our previous work [47] that suggests that greater quadriceps RTD symmetry in females post-ACLR is associated with more symmetrical double-leg landing mechanics, but the quadriceps RTD magnitude of the ACLR limb is not.

Overall, the results of the current study suggest that evaluating and improving explosive quadriceps muscle strength during rehabilitation and before return to play following ACLR, and in female without ACLR can facilitate the use of a safer landing mechanics. Such findings have clinical implication for injury prevention. We recommend clinicians should include intervention aimed at increasing explosive muscle strength such as whole body vibration training [48] and plyometric training [49] to facilitate quadriceps RTD for preventing ACL injury and post-traumatic knee osteoarthritis post-ACLR.

There are limitations to consider when interpreting the results of this study. The current study only investigated sagittal plane landing mechanics of the knee. Given that ACL injuries likely occur due to a multi-joint coordination and multi-planar mechanism of injury [19], future studies that evaluate the relationship of quadriceps RTD and potential compensatory movement patterns on frontal- and transverse-plane biomechanics at the ankle, knee and hip joints and are needed. Another limitation is that the current study did not limit the graft type and a specific surgeon. However, regardless of graft type or surgeon, individuals who wish to return to sport are likely performing the athletic tasks such as landing and cutting after return following ACLR. In order to maximize the generalizability of our study, we chose not to limit graft types and a specific surgeon.

The fact that standardized footwear was not used is one of the limitations of the study. However, we chose not to control for shoes type because while controlling for shoe type standardizes the mechanical properties of the shoe, it introduces potential errors in that the use of an unfamiliar shoe could affect participants’ nature landing mechanics [26]. The second rationale that we chose to have participants wear their own athletic shoes is to maximize the study findings’ generalizability. The choice of not controlling for athletic footwear allows us to observe landing mechanics that better represent real-world situations and, therefore, increase the study’s external validity. Given there were no group differences and group*task interaction was found in the study, any effect would of shoe would only confound the results if there was a systematic bias for low RTD participants to have one type of shoe and high RTD participants to have another, which we did not observe during data collection. Lastly, our ACLR group exhibited a wide range of time post-ACLR (35.1 ± 13.7 months) and they had participated in a high level of physical activity. Therefore, less active females’ post-ACLR could exhibit a different movement biomechanics profile and muscle function.

## 5. Conclusions

The current study found that greater quadriceps RTD is associated with a lower loading rate by lengthening the time to peak impact during single-leg jump and cutting maneuvers regardless of the ACLR status. Greater loading rate may lead to higher risk of ACL injury and post-traumatic knee osteoarthritis post-ACLR. Thus, to facilitate the use of safer landing mechanics, explosive muscle strength interventions might be useful for individuals with and without ACLR to improve quadriceps RTD.

## Figures and Tables

**Table 1:** Participant Demographics and Group Comparisons.

Characteristics	Control (*N* = 12) ^a^	ACLR (*N* = 18) ^a^	*p* Value
Age (year)	21.0 ± 2.6	19.9 ± 1.2	0.12
Height (cm)	163.5 ± 7.7	165.1 ± 6.4	0.55
Mass (kg)	57.3 ± 6.5	63.8 ± 11.0	0.08
Tegner Activity Scale	6.6 ± 0.1	6.4 ± 1.5	0.59
Quadriceps RTD (Nm × s^−1^ × kg^−1^) ^c^	13.4 ± 5.4	13.5 ± 4.9	0.94
Time after surgery (month)	-	35.1 ± 13.7	-

^a^ Mean ± SD; ^c^ Onset to 100 milliseconds after onset; RTD: rate of torque development.

**Table 2:** Landing biomechanics during double-leg jump landings.

Variables	Control (*N* = 12) ^a^	ACLR (*N* = 18) ^a^
Knee flexion at IC (°)	18.0 ± 6.3	16.2 ± 4.1
Knee flexion excursion (°)	72.2 ± 10.4	72.3 ± 8.2
Peak knee flexion (°)	90.2 ± 6.7	88.5 ± 8.6
Peak knee extension moment (N × m × N^−1^)	−0.4 ± 0.1	−0.4 ± 0.1
Peak vGRF (N × N^−1^)	3.1 ± 0.4	3.2 ± 0.6
Peak pGRF (N × N^−1^)	−0.8 ± 0.1	−0.8 ± 0.1
Peak ATSF (N × N^−1^)	1.2 ± 0.2	1.3 ± 0.2
Time to peak knee extension moment (ms)	85.4 ± 6.8	85.5 ± 6.3
Time to peak vGRF (ms)	56.9 ± 7.1	61.0 ± 8.0
Time to peak pGRF (ms)	69.5 ± 16.3	62.8 ± 15.6
Time to peak ATSF (ms)	90.8 ± 7.6	86.1 ± 8.0
Rate of knee extension moment (N × m × N^−1^ × s^−1^)	−4.4 ± 1.1	−4.5 ± 0.7
Loading rate ((N × N^−1^) × s^−1^)	55.1 ± 11.7	53.2 ± 13.2

^a^ Mean ± SD; IC: initial contact, vGRF: vertical ground reaction force, pGRF: posterior ground reaction force, ATSF: anterior tibial shear force.

**Table 3:** Landing biomechanics during single-leg jump cuts.

Variables	Control (*N* = 12) ^a^	ACLR (N = 18) ^a^
Knee flexion at IC (°)	22.2 ± 7.3	20.6 ± 6.6
Knee flexion excursion (°)	38.2 ± 7.8	37.9 ± 7.3
Peak knee flexion (°)	60.4 ± 5.8	58.5 ± 6.9
Peak knee extension moment (N × m × N^−1^)	−0.5 ± 0.1	−0.5 ± 0.1
Peak vGRF (N × N^−1^)	5.1 ± 0.4	5.0 ± 0.6
Peak pGRF (N × N^−1^)	−0.6 ± 0.1	−0.6 ± 0.2
Peak ATSF (N × N^−1^)	2.2 ± 0.2	2.2 ± 0.3
Time to peak knee extension moment (ms)	97.4 ± 2.6	96.5 ± 4.5
Time to peak vGRF (ms)	80.0 ± 14.0	82.7 ± 13.9
Time to peak pGRF (ms)	69.9 ± 18.4	61.0 ± 19.3
Time to peak ATSF (ms)	98.8 ± 1.0	98.1 ± 3.1
Rate of knee extension moment (N × m × N^−1^ × s^−1^)	−5.0 ± 0.7	−4.9 ± 0.5
Loading rate ((N × N^−1^) × s^−1^)	65.4 ± 15.0	61.7 ± 13.0

^a^ Mean ± SD; IC: initial contact, vGRF: vertical ground reaction force, pGRF: posterior ground reaction force, ATSF: anterior tibial shear force.
